# Additive Contributions of Two Manganese-Cored Superoxide Dismutases (MnSODs) to Antioxidation, UV Tolerance and Virulence of *Beauveria bassiana*

**DOI:** 10.1371/journal.pone.0030298

**Published:** 2012-01-18

**Authors:** Xue-Qin Xie, Fang Li, Sheng-Hua Ying, Ming-Guang Feng

**Affiliations:** Institute of Microbiology, College of Life Sciences, Zhejiang University, Hangzhou, Zhejiang, People's Republic of China; Instituto de Biociencias - Universidade de São Paulo, Brazil

## Abstract

The biocontrol potential of entomopathogenic fungi against arthropod pests depends on not only their virulence to target pests but tolerance to outdoor high temperature and solar UV irradiation. Two *Beauveria bassiana* superoxide dismutases (SODs), BbSod2 and BbSod3, were characterized as cytosolic and mitochondrial manganese-cored isoenzymes (MnSODs) dominating the total SOD activity of the fungal entomopathogen under normal growth conditions. To probe their effects on the biocontrol potential of *B. bassiana*, Δ*BbSod2*, Δ*BbSod3*, and three hairpin RNA-interfered (RNAi) mutants with the transcripts of both *BbSod2* and *BbSod3* being suppressed by 91–97% were constructed and assayed for various phenotypic parameters in conjunction with Δ*BbSod2*/*BbSod2*, Δ*BbSod3/BbSod3* and wild-type (control strains). In normal cultures, the knockout and RNAi mutants showed significant phenotypic alterations, including delayed sporulation, reduced conidial yields, and impaired conidial quality, but little change in colony morphology. Their mycelia or conidia became much more sensitive to menadione or H_2_O_2_-induced oxidative stress but had little change in sensitivity to the hyperosmolarity of NaCl and the high temperature of 45°C. Accompanied with the decreased antioxidative capability, conidial tolerances to UV-A and UV-B irradiations were reduced by 16.8% and 45.4% for Δ*BbSod2*, 18.7% and 44.7% for Δ*BbSod3*, and ∼33.7% and ∼63.8% for the RNAi mutants, respectively. Their median lethal times (LT_50_s) against *Myzus persicae* apterae, which were topically inoculated under a standardized spray, were delayed by 18.8%, 14.5% and 37.1%, respectively. Remarkably, the effects of cytosolic BbSod2 and mitochondrial BbSod3 on the phenotypic parameters important for the fungal bioncontrol potential were additive, well in accordance with the decreased SOD activities and the increased superoxide levels in the knockout and RNAi mutants. Our findings highlight for the first time that the two MnSODs co-contribute to the biocontrol potential of *B. bassiana* by mediating cellular antioxidative response.

## Introduction

Superoxide dismutases (SODs) are metalloproteins that can scavenge intracellular reactive superoxide species (ROS) and serve as the first-line enzymes in cellular defense system against superoxide damage [1]. Like most eukaryotes, fungi possess cytosolic Cu/ZnSODs and homotetrameric MnSODs, which were found early in mitochondrial matrix [2] and later in cytosol [3] and peroxisome [4]. Indeed, two different MnSODs co-exist in the cytosol and mitochondria of human pathogen *Candida albicans* [5] and several fungi [6].

Fungal SODs are involved in various biological processes including stress response, cell differentiation and infection [7]. For instance, mitochondrial MnSOD protects yeast cells from the damages of hyperosmolarity, heat and oxidation [8], 9,10,11. A null mutant of *Aspergillus fumigates* mitochondrial MnSOD could not grow under thermal and oxidative stresses while the cytosolic counterpart showed slightly delayed growth at high temperature [12]. The inactivation of a fungal or yeast SOD resulted in reduced mycelial growth [13], disordered conidiation rhythm [14], decreased sporulation potential [15], and delayed conidial germination [12]. Moreover, the MnSOD-disrupted mutants of *A. fumigates* and *Cryptococcus neoformans* became less virulent to mice [10], [12]. However, the pathogenicity of *Colletotrichum graminicola* was not altered by disrupted MnSOD due to possible complementary effects from two other isoenzymes in the phytopathogenic fungus [3].

*Beauveria bassiana* is a typical entomopathogenic fungus that infects not only a wide spectrum of insect pests but also mites and ticks. The fungal biocontrol potential depends not only on the virulence of a candidate strain to target pests but also on its tolerance to high temperatures and solar UV irradiations often encountered in the field [16]. Two SODs have recently been identified from *B. bassiana* [17], [18], including Cu/ZnSOD (BbSod1) and MnSOD (BbSod2) with no mitochondria-targeting signal. Of those, the cytosolic BbSod2 was found contributing significantly to antioxidative capability, UV-B resistance and virulence of *B. bassiana* when it was overexpressed in a wild-type strain lacking the enzyme [18]. Although biological functions of individual MnSODs have been characterized in some human or plant pathogenic fungi, the effects of different SODs and their interactions on the biocontrol potential of fungal entomopathogens remain poorly understood. Since BbSod2 and another mitochondrial MnSOD (BbSod3) were revealed as isoenzymes dominating the total SOD activity of *B. bassiana*, this study sought to characterize the functions of the two different MnSODs and distinguish their contributions to the fungal biocontrol potential by constructing the knockout and complement mutants of each enzyme and the mutants of both enzymes silenced by hairpin RNA interference (RNAi) and evaluating various phenotypic alterations under normal and stressful conditions.

## Results

### Features of *BbSod3* and the deduced protein

**Figure 1:**
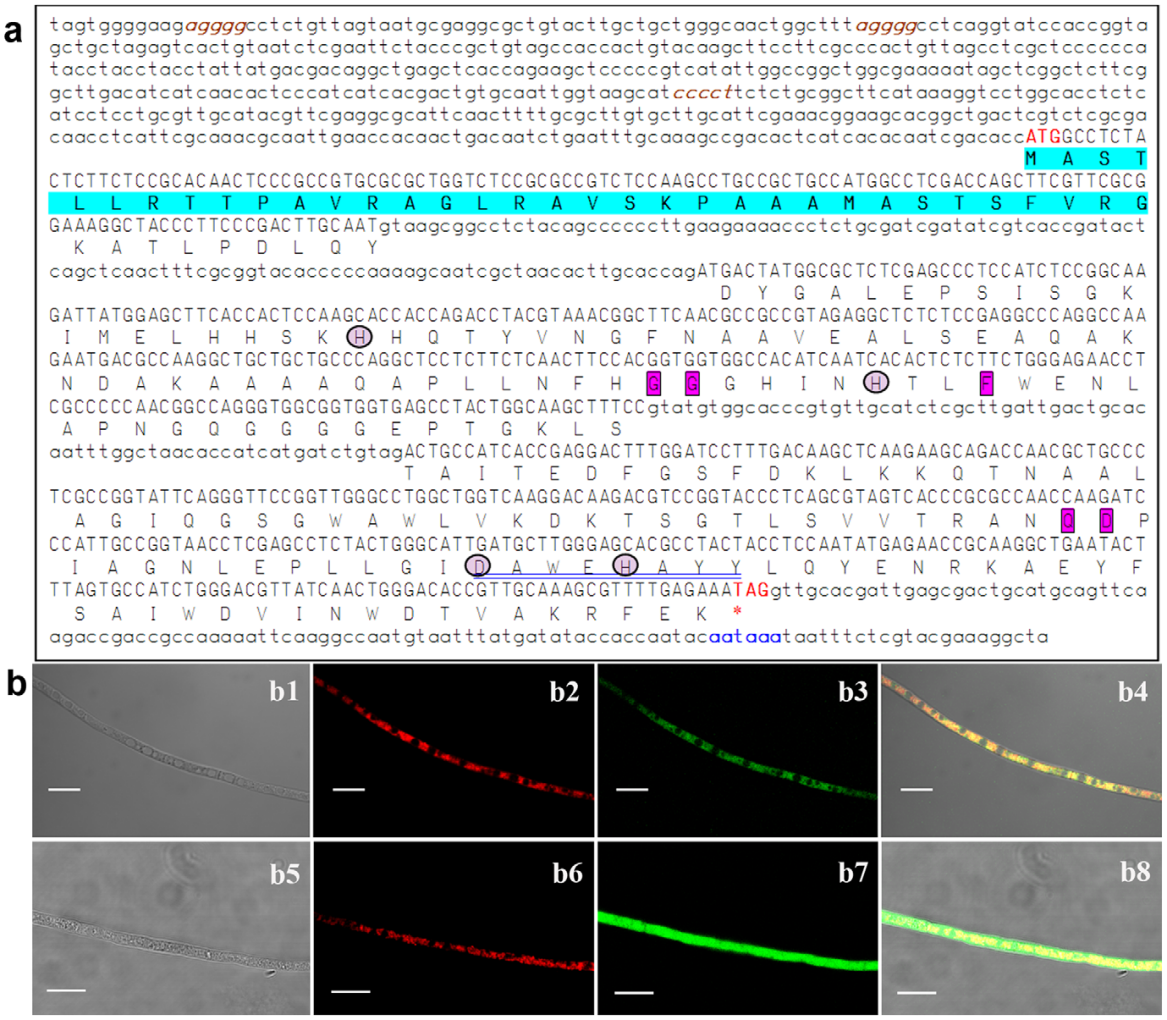
The features of *BbSod3* encoding mitochondrial MnSOD in *B. bassiana*. (**a**) The nucleotide and deduced protein sequences of *BbSod3*. The uppercase DNA fragment is a 693-bp ORF encoding a protein of 230 amino acids while the lowercase fragments are ORF-flanking regions. Located in 5′ UTR and 3′ UTR are three putative stress-response elements (brown and italisized) and a putative polyadenylation signal (blue), respectively. Note that the first 34 amino acids (highlighted) of the deduced protein were predicted as a mitochondria-targeted signal peptide. The framed residues are the Parker and Blake signatures typical for the Mn-SOD family and the circled residues are metal-binding sites. The sequence double-underlined in blue represents the consensus pattern DXWEHXXY for the Fe/Mn-SOD superfamily. (**b**) Intracellular localization of BbSod3. Mycelia of transgenic strains expressing the fusion BbSod3signal::eGFP (b1–b4) and the signal-free eGFP (b5–b8, control) were stained with the mitochondria-probing stain MitoTracker® Red, emitting the red (stain) and green (eGFP) fluorescences. The differential interference contrast image (b1) and the same image labeled with the stain (b2) and the expressed fusion (b3) overlapped very well, forming the merged image (b4) whose color pattern (entirely brown-yellow in reticulum components) indicates the mitochondrial target of the fused signal peptide, whereas the merged control image (b8) showed more green than yellow. Scale bars: 10 µm.

A 220-bp fragment was amplified from the genomic DNA of a *B. bassiana* wild-type strain (WT) via PCR with degenerate primers (Table 1). The full-length *BbSod3* gene (GenBank ID: HM030722) with the flanking regions obtained by DNA walking consisted of a 693-bp ORF and two introns (186 bp in total), as illustrated in Fig. 1a. The exon-intron boundaries were characteristic with a basic consensus (GT/AG) for eukaryotic splice donor and acceptor sites [19]. An internal putative splice box, i.e., CTRAY, was located upstream of the 3′ ends of both introns. Three stress-responsive elements (AGGGG or CCCCT) and one typical eukaryotic polyadenylation signal sequence (AATAAA) were found in the up- and downstream regions, respectively, but no typical TATA box was present in the upstream region.

**Table 1:** The paired primers used for gene cloning, disruption, complement, silencing and expression.

Paired primers	Sequences (5′-3′) *	Purpose
Sod3-F/R	TATGACTATGGHGCBCTBGA/GCVAGGTTCTCCCARAAVAG	*Sod3* domain cloning
Sod3-upR1/R2	CAGCAGCCTTGGCGTCATTC/GGTCTGGTGGTGCTTGGAGTG	DNA walking
Sod3-dnF1/F2	ACCACTCCAAGCACCACCAG/GGCTCCTCTTCTCAACTTCCACG	DNA walking
Sod3-F1/R1	ATGGCCTCTACTCTTCTCCGCAC/CTATTTCTCAAAACGCTTTGCAACG	Full-length *Sod2* cloning
SigGFP-F1/R1	GA*AGATCT* TATGGCCTCTACTCTTCTCCGCAC/GCTCCTCGCCCTTGCTCACTCCGCGAACGAAGCTGGTCG	Signal cloning
SigGFP-F2/R2	CGACCAGCTTCGTTCGCGGAGTGAGCAAGGGCGAGGAGC/GA*AGATCT*TTACTTGTACAGCTCGTCCATGC	eGFP cloning
PtrpC-F/R	CCC*AAGCTT*CGACGTTAACTGATATTGAAGGAGC/CGGGCGTCGTTCTGGGCTCATTTGGATGCTTGGGTAGAATAGG	P*trpC* cloning
Bar-F/R	CCTATTCTACCCAAGCATCCAAATGAGCCCAGAACGACGCCCG/GA*AGATCTTCTAGACTCGAGT*CAGATCTCGGTGACGGGCAGGAC	*bar* cloning
L1/L2	CCG*GAATTC*CTGGATAATGACGCAAAGGTCC/CGCGGATCCAGAGCGAGTGGTTGATGTGGC	5′ *sod2* cloning
R1/R2	CCG*CTCGAG*GACAGAATCGCAGTACCTCAACG/GG*ACTAGT*GGCTGGACAAGACGGAACTGC	3′ *sod2* cloning
L3/L4	CGC*GGATCC*AGGACCTGGCTCGGGCGTATC/AAAA*CTGCAG*TCTGGTGCAAGTGTTAGCGATT	5′ *sod3* cloning
R3/R4	AAGC*TCTAGA*GACTGCCATCACCGAGGACTT/GG*ACTAGT*AAAGCAAGCCTGTTAGCAAATC	3′ *sod3* cloning
Sur-F/R	CG*GAATTC*GTCGACGTGCCAACGCCACAGTG/CG*GGATCC*GTCGACGTGAGAGCATGCAATTCC	*Sur* cloning
Sod2C-F/R	**GGGGACCACTTTGTACAAGAAAGCTGGGT**CACAGTCAGGAGAGAAGAGA/**GGGGACAAGTTTGTACAAAAAAGCAGGCT**GAAGAGTTTTGATAGATGGGAG	Cloning full-length *Sod2*
Sod3C-F/R	**GGGGACCACTTTGTACAAGAAAGCTGGGT**AGGAGACGGTCGAGACTCTG/**GGGGACAAGTTTGTACAAAAAAGCAGGCT**GTTCTCGCAGCGGTTTCAATC	Cloning full-length *Sod3*
I1/I2	ACATCACCGCCCAAATCGCCCTC/ACGCTCAGCCGTCTTCCAGTTG	PCR for Δ*BbSod2*
I3/I4	TACCCTTCCCGACTTGCAATGTAAG/TGCGGTTCTCATATTGGAGGTAG	PCR for Δ*BbSod3*
I-F/I-R	CATG*CCATGGACTAGTGTTTAAAC*GATCGAGAATACCATCACC/CG*GGATCCGAATTCGATATC*CCAGACGGAACGGCAGAG	Loop cloning
F1sen-F1/R1	CATG*CCATGG*ATGGCTCACAACTACTCGCTCC/CAAGTCGGGAAGGGTAGCCTTGATGGACGCCTTGAGAGCCTTG	*F1*-sense repeat
F1sen-F2/R2	CAAGGCTCTCAAGGCGTCCATCAAGGCTACCCTTCCCGACTTG/GG*ACTAGT*CGGTGATGGCAGTGGAAAGC	*F1*-sense repeat
F1anti-F/R	CCG*GAATT*CGGTGATGGCAGTGGAAAGC/GGA*AGATCT*ATGGCTCACAACTACTCGCTCC	*F1*-antisense repeat
F1sen-F1/F2sen-R1	CATG*CCATGG*ATGGCTCACAACTACTCGCTCC/CAAAGTCCTCGGTGATGGCAGATGGACGCCTTGAGAGCCTTG	*F2*-sense repeat
F2sen-F2/R2	CAAGGCTCTCAAGGCGTCCATCTGCCATCACCGAGGACTTTG/GG*ACTAGT*CTATTTCTCAAAACGCTTTGCAACG	*F2*-sense repeat
F2anti-F/F1anti-R	CCG*GAATTC*CTATTTCTCAAAACGCTTTGCAACG/GGA*AGATCT*ATGGCTCACAACTACTCGCTCC	*F2*-antisense repeat
18S-F/R	TGGTTTCTAGGACCGCCGTAA/CCTTGGCAAATGCTTTCGC	qRT-PCR of 18S rRNA
Sod2RT-F/R	CCAGTGTTTGGCATTGACATG/TCAGCCGTCTTCCAGTTGATG	qRT-PCR of *BbSod2*
Sod3RT-F/R	ACATCAATCACACTCTCTTCTG/GCGTTGGTCTGCTTCTTG	qRT-PCR of *BbSod3*

*H = A, C or T; B = C, G or T; V = A, C or G; R = A or G. The enzyme sites are italicized while the gateway fragments for exchange are in bold.

The ORF was deduced to encode a protein sequence of 230 amino acids with a predicted molecular weight of 24.7 kDa and an isoelectric point of 7.6. The deduced protein was characteristic with a conserved sequence (DAWEHAYY) pattern typical for the Mn/FeSOD superfamily [20], the residues His64, His108, Asp194 and His198 for metal binding, and the MnSOD signatures of Gly102, Gly103, Phe111, Gln180 and Asp181 [21]. Moreover, BbSod3 was found sharing 36–94% sequence identity with other 75 fungal MnSODs in the NCBI protein database. Interestingly, only 47% sequence identity was found between BbSod3 and BbSod2, a cytosolic MnSOD characterized previously from the same strain [18]. Pfam analysis showed the presence of C-terminal α-hairpin domain (typical for Mn/FeSODs) in the deduced BbSod3. The first 34 amino acids of BbSod3 were predicted as a mitochondria targeting signal sequence at the probability of 0.9987. The putative signal sequence was similar to those for mitochondrial transports, i.e., the sequences of 20–80 amino acids in which 3–5 lysine or arginine residues are separated by 2–5 hydrophobic residues from one to another [22]. All the analyses indicated that BbSod3 was a new mitochondrial MnSOD.

The predicted subcellular location for BbSod3 was further confirmed using transgenic strains expressing a fusion of the signal peptide and enhanced green fluorescent protein (eGFP), which was used to localize the protein targeted by the signal peptide. Thirty-five of 40 transgenic strains examined under fluorescence microscope emitted green fluorescence, which resulted from the BbSod3signal::eGFP expressed in mycelia. The green fluorescence distributed in intracellular reticular components overlapped very well with the red fluorescence from mycelial mitochondria stained with the probe MitoTracker Red (Fig. 1, b1–b4). In contrast, the green fluorescence was evenly distributed in the entire mycelia of a strain expressing the signal-free eGFP (Fig. 1, b5–b8). These data indicated that the signal peptide directed the target protein towards mitochondria. Therefore, BbSod3 was confirmed as a mitochondrial MnSOD.

### Distinctive SOD-active band for BbSod3 versus BbSod2

**Figure 2:**
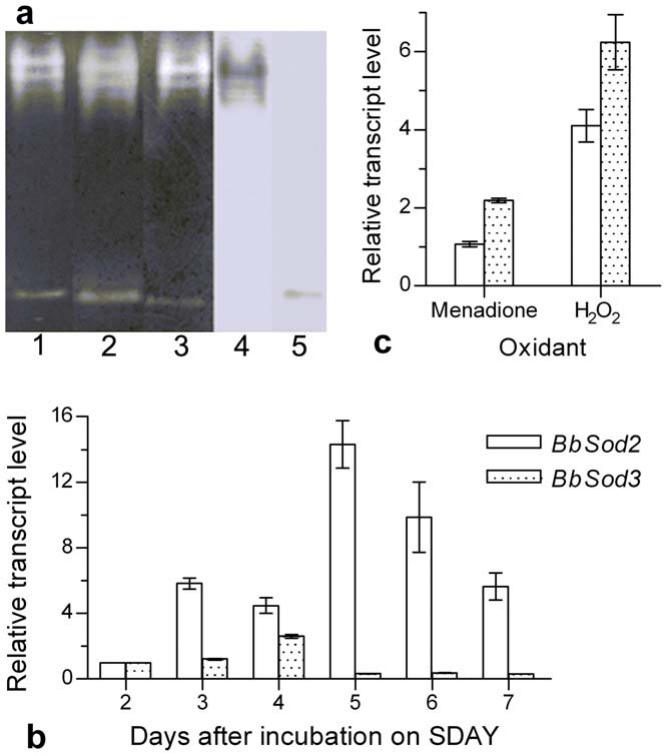
The SOD enzymograph of BbSod2 and BbSod3 and their gene transcripts in *B. bassiana* wild-type (WT). (**a**) SOD-active bands on the Native-PAGE gels stained with NBT only (Lane 1) or together with KCN (Cu/ZnSOD-specific inhibitor, Lane 2) or H_2_O_2_ (FeSOD-specific inhibitor, Lane 3), or blotted with the polyclonal antibodies anti-BbSod2 (Lane 4) and anti-BbSod3 (Lane 5). All the lanes were uploaded with the protein extract samples from 3-day colonies (mycelia) grown on SDAY at 25°C. (**b**) Transcript levels (relative to day 2) during 7-day growth on SDAY plates at 25°C, determined by qRT-PCR. (**c**) Transcript levels (relative to unstressed control) in the colonies grown at 25°C for 3 days on SDAY under the oxidative stress of 0.2 mM menadione or 4 mM H_2_O_2_. Error bars: SD of the mean from three replicates.

Two distinguished SOD-active bands (Fig. 2a, lanes 1–3) were present on all NBT-stained Native PAGE gels uploaded with WT protein samples irrespective of KCN, H_2_O_2_ or none of both SOD inhibitors added to the phosphate buffer for 20 min incubation prior to staining. This demonstrated that the two bands resulted from the activities of MnSODs. Western blots probed with anti-BbSod2 and anti-BbSod3 (Fig. 2a, lanes 4 and 5) confirmed that the upper and lower bands were BbSod2 and BbSod3, respectively. Apparently, cytosolic BbSod2 and mitochondrial BbSod3 dominated the overall SOD activity of *B. bassiana* under normal growth conditions.

### Transcript pattern of *BbSod3* versus *BbSod2*

In normal WT cultures initiated by spreading conidia on SDAY and grown at 25°C for 7 days, *BbSod3* transcript levels were 20.8%, 58.3%, 2.2%, 3.7% and 5.3% of those measured for *BbSod2* on days 3–7 (Fig. 2b), respectively, based on the quantitative real-time PCR (qRT-PCR) analysis of total RNAs extracted from the cultures. In contrast, the *BbSod3* transcripts in the 3-day cultures under the oxidative stresses of 0.2 mM menadione and 4 mM H_2_O_2_ were increased by 105% and 52% compared with the *BbSod2* counterparts (Fig. 2c).

### Disruption and complement mutants of *BbSod2* and *BbSod3*

**Figure 3:**
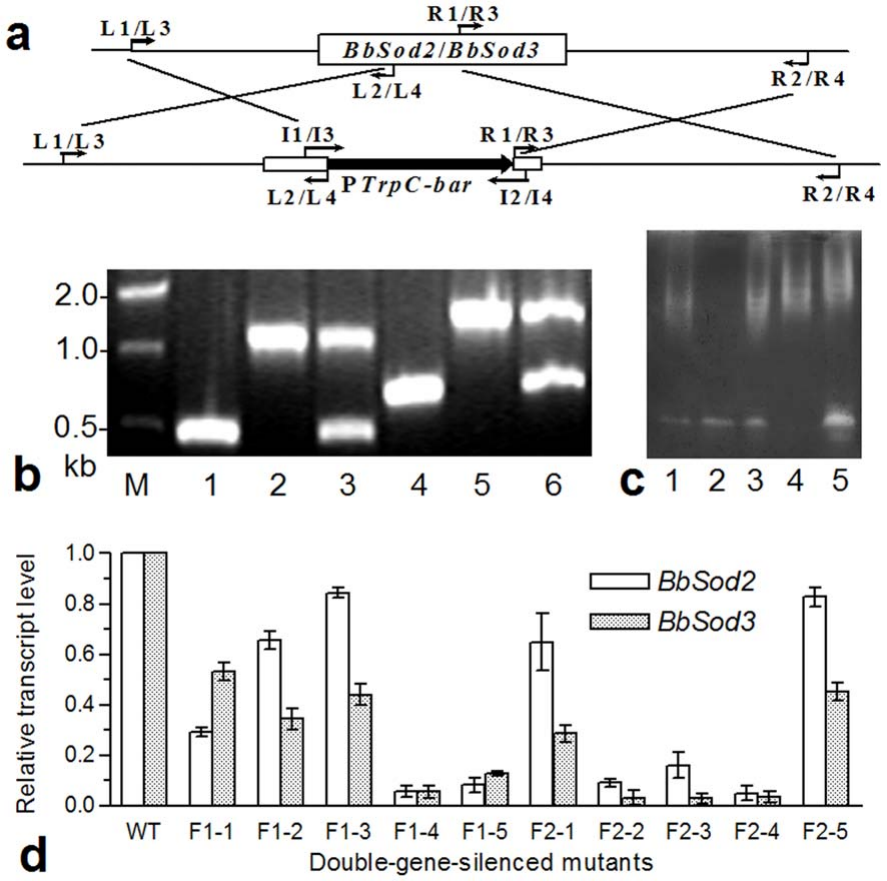
Knockout and complement of BbSod2 and BbSod3 and RNAi double silence of both enzymes. (**a**) Diagram for the knockout constructs of *BbSod2* and *BbSod3* (see Table 1 for the used primers). (**b**) Detection of the disrupted and complemented *BbSod2* and *BbSod3* fragments by PCR with paired primers I1/I2 (Lanes 1–3: WT, Δ*BbSod2* and Δ*BbSod2*/*BbSod2*) and I3/I4 (Lanes 4–6: WT, Δ*BbSod3* and Δ*BbSod3*/*BbSod3*). (**c**) SOD active bands on the NBT-stained gels of WT (Lane 1) and mutants (Lanes 2–5: Δ*BbSod2*, Δ*BbSod2*/*BbSod2*, Δ*BbSod3* and Δ*BbSod3*/*BbSod3*, respectively). (**d**) RNAi double silence. Relative transcript levels of *BbSod2* and *BbSod3* in 10 RNAi mutants of the fused genes *F1* and *F2* were assessed via qRT-PCR. Both fusions were constructed with *BbSod2* ORF and partial *BbSod3* ORF. Note that the desired double-gene silence was achieved in the mutants F1-4, F2-2 and F2-4. Error bars: SD of the mean from three replicates.

*BbSod2* and *BbSod3* were successfully disrupted by replacing partial ORF regions (341 and 334 bp, respectively) with the P*trpC*-*bar* cassette of ∼900 bp (Fig. 3a) and complemented by integrating each gene into its knockout mutant. The PCR analysis with paired primers showed the single band of ∼1.2 or ∼1.3 kb in the profile of Δ*BbSod2* or Δ*BbSod3* and of ∼0.5 or ∼0.7 kb in the WT profile (Fig. 3b). In contrast, both bands were present in the profile of each complement mutant. All the constructs were well confirmed by the presence or absence of the SOD bands on the NBT-stained gels (Fig. 3c).

### RNAi mutants of two *BbSod2::BbSod3* fusions

Five RNAi mutants of each *BbSod2::BbSod3* fusion (*F1* or *F2*) randomly taken from selective Czapek's plates were grown on SDAY plates. The qRT-PCR analysis indicated that the transcripts of *BbSod2* and *BbSod3* in the 3-day SDAY cultures of all RNAi mutants (Fig. 3d) were suppressed by 20–97% (*BbSod2*: *F*_10,22_ = 213, *P*<0.0001; *BbSod3*: *F*_10,22_ = 314, *P*<0.0001). Of those, three mutants, i.e., F1-4, F2-2 and F2-4, showed the expected double silence of both target genes. The transcript levels of *BbSod2* and *BbSod3* in their cultures were equal to only 5.1–9.2% and 3.1–5.6% of the WT transcripts, respectively. Thus, the three RNAi mutants were chosen as desired double-gene-silenced mutants for the following triplicate biochemical and phenotypic assays in conjunction with the null mutants and the control strains WT, Δ*BbSod2/BbSod2* and Δ*BbSod3/BbSod3*.

### SOD activities and ROS levels in knockout and RNAi mutants

**Figure 4:**
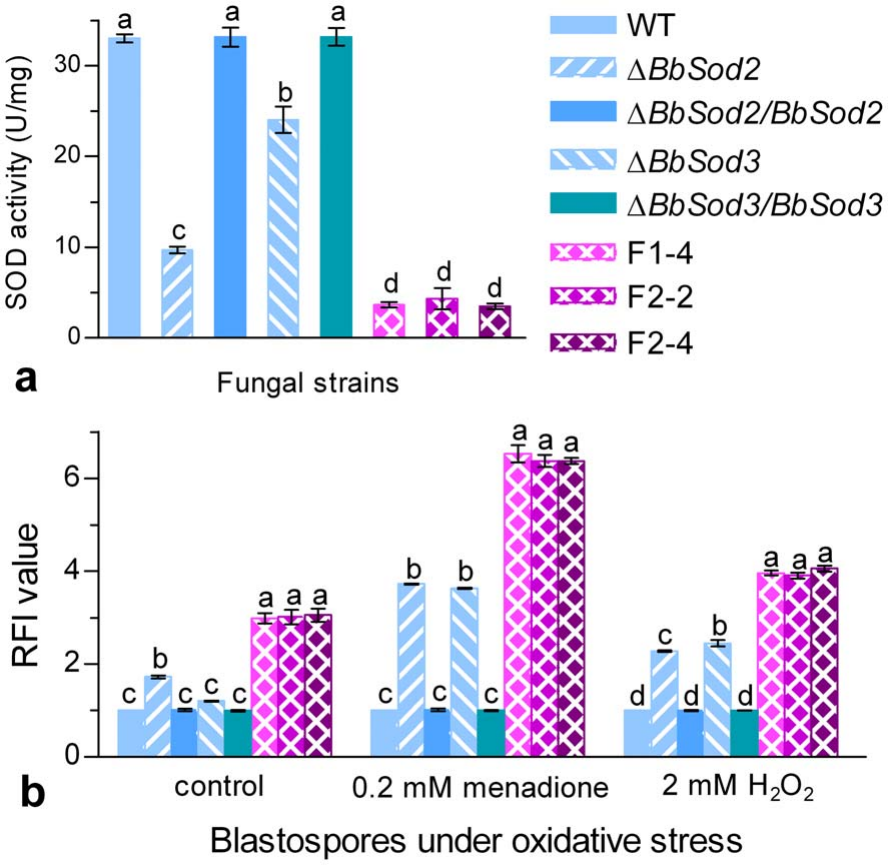
Overall SOD activities and ROS levels in the cells of WT and mutant strains. (**a**) SOD activities in protein extracts from 3-day cultures on SDAY at 25°C. (**b**) Relative fluorescence intensity (RFI) values of the blastospores stained with the fluorescence probe dihydroethidium (DHE). The thin-walled, unicellular blastospores produced in a nitrogen-limited medium (NLM) were resuspended in NLM containing 0.2 mM menadione, 2 mM H_2_O_2_ or no oxidant (control) for 1 h incubation prior to staining. Each RFI value indicates the intracellular ROS level of each mutant versus WT under a given condition. Different lowcase letters on the bars of each group denote significanct differences (Tukey's HSD, *P*<0.05). Error bars: SD of the mean from three replicates.

Illustrated in Fig. 4a are the total SOD activities in the normal cultures of all mutants versus WT. As a result of the drastic down-regulation of both *BbSod2* and *BbSod3* in the three RNAi mutants, their SOD activities were reduced by 55–64%, 82–85% and 87–89% compared with those from Δ*BbSod2* (9.7 U/mg proteins), Δ*BbSod3* (24.0 U/mg) and the control strains (33.0–33.2 U/mg), respectively, but did not differ significantly from one to another (Tukey's HSD, *P*≥0.93). These data indicated that cytosolic BbSod2 contributed much more to the total SOD activity than mitochondrial BbSod3 due to the loss of 71% SOD activity in Δ*BbSod2* and of 27% in Δ*BbSod3* under the normal culture conditions.

Moreover, as indicated by the relative fluorescence intensity (RFI) of the blastospores cultured in a nitrogen-limited medium (NLM) and then stained with dihydroethidium (DHE) as fluorescence probe, intracellular ROS level increased by 72% in Δ*BbSod2* and ∼2 fold in three RNAi mutants compared with the RFI values from the control strains (Fig. 4b), but the increase was insignificant for Δ*BbSod3* (Tukey's HSD, *P*≥0.13). Inclusion of 0.2 mM menadione or 2 mM H_2_O_2_ in the NLM cultures for 1 h incubation resulted in higher ROS levels in all knockout and RNAi mutants, which responded stronger to menadione than H_2_O_2_. Interestingly, Δ*BbSod3* showed similar or slightly higher ROS level than Δ*BbSod2* in response to the two oxidants.

### Sporulation capacity and conidial quality of knockout and RNAi mutants

**Figure 5:**
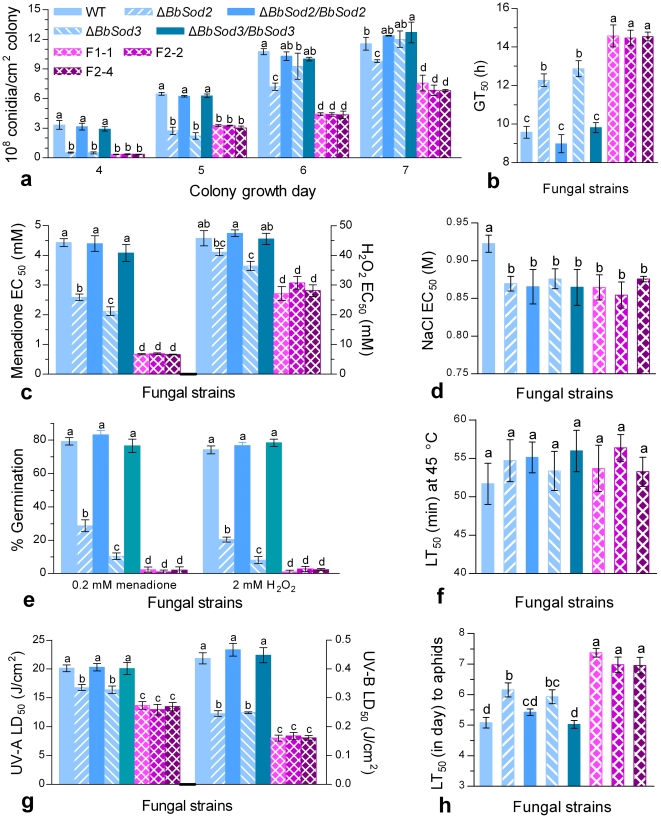
Phenotypic parameters of knockout and RNAi mutants of BbSod2 and BbSod3 versus wild-type (WT). (**a**) Conidial yields in SDAY colonies grown for 4–7 days at 25°C. (**b**) Time length (GT_50_) required for 50% conidial germination under normal conditions. (**c**), (**d**) Effective concentration (EC_50_) for H_2_O_2_, menadione or NaCl to suppress 50% colony growth. (**e**) Percent germination of conidia under the oxidative stress of menadione or H_2_O_2_. (**f**) Median lethal time (LT_50_) for conidial tolerance to the thermal stress at 45°C. (**g**) Median lethal dose (LD_50_) for conidial tolerance to UV-A or UV-B irradiation. (**h**) LT_50_ for conidial virulence to *M. persicae* adults. Different lowcase letters on the bars of each group denote significanct differences (Tukey's HSD, *P*<0.05). Error bars: SD of the mean from three repeated assays.

All fungal colonies were similar in color and size during 7-day growth on SDAY plates at 25°C irrespective of WT or mutant strains. Under the normal conditions, the control strains started sporulation on day 3 and produced significantly more conidia than the knockout and knockdown mutants on days 4–6 despite diminished differences on the last day (Fig. 5a). The final conidial yields of the three double-silenced mutants were reduced by 23–31%, 37–43% and 35–46% compared with those measured from Δ*BbSod2*, Δ*BbSod3* and the control strains, respectively. These data indicated that both BbSod2 and BbSod3 affected the sporulation rhythm and potential of *B. bassiana* but BbSod2 was more influential.

Moreover, temporal patterns of conidial germinations on the plates of germination medium (GM) varied greatly among the tested strains although their germinations reached 93–99% after 24-h incubation at 25°C. As a result of fitting the temporal patterns, the time (GT_50_) required for 50% germination was delayed by 2.8 h for Δ*BbSod2*, 3.4 h for Δ*BbSod3*, and 5.1 h for the RNAi mutants compared with the mean GT_50_ (9.5 h) from the control strains (Fig. 5b). This indicated that conidial quality was significantly impaired by the disruption of either enzyme gene and much more by the silence of both genes (*F*_7,16_ = 114, *P*<0.0001).

### Colony tolerance to oxidation and hyperosmolarity

During 7-day growth on 1/4 SDAY plates at 25°C, Δ*BbSod2* and Δ*BbSod3* were more sensitive to the oxidative stress of either menadione or H_2_O_2_ than the control strains but less sensitive than the three RNAi mutants (Fig. 5c). The effective concentration (EC_50_) for menadione to suppress 50% colony growth decreased 42% for Δ*BbSod2* (2.6 mM), 52% for Δ*BbSod3* (2.1 mM) and ∼85% for the RNAi mutants (0.66–0.68 mM) compared with the EC_50_s of 4.1–4.4 mM from the control strains. The H_2_O_2_ EC_50_ was estimated as 42.2 mM for Δ*BbSod2*, 38.2 mM for Δ*BbSod3*, and 29.7–32.2 mM for the RNAi mutants. These estimates were averagely 12.6%, 21.0% and 36.5% lower than the control estimates of 47.6–48.6 mM, respectively. Overall, BbSod2 and BbSod3 contributed additively to the fungal antioxidative capability during colony growth.

All knockout, complement and knockdown mutants tended to equally respond to the hyperosmotic stress of a given NaCl concentration during the growth on 1/4 SDAY (Fig. 5d) although they were seemingly more sensitive to the stress than WT (*F*_7,16_ = 4.9, *P* = 0.004).

### Conidial tolerance to oxidation, UV irradiation and heat

Under the oxidative stress of 0.2 mM menadione added to the GM plates, the conidial germinations of Δ*BbSod2*, Δ*BbSod3* and three RNAi mutant (Fig. 5e) were observed as 28.7%, 10.4%, and 1.0–2.3%, respectively, while the same observations from the control strains fell in the range of 77–83%. Inclusion of 2 mM H_2_O_2_ in the plates resulted in similar changes in percent germinations. Overall, under the stresses of menadione and H_2_O_2_, conidial germinations were reduced by 64% and 73% for Δ*BbSod2*, 87% and 89% for Δ*BbSod3*, and ∼97% for the RNAi mutants, respectively.

Median lethal doses (LD_50_s) estimated by fitting the inverted sigmoid trends of surviving conidia over the gradient doses of UV-A and UV-B irradiations differed significantly among the tested strains (UV-A: *F*_7,16_ = 55, *P*<0.0001; UV-B: *F*_7,16_ = 227, *P*<0.0001). For all the tested strains, conidia were far more sensitive to UV-B (LD_50_s: 0.16–0.47 J/cm^2^) than UV-A (LD_50_s: 13.0–20.3 J/cm^2^). Compared with the LD_50_s from the control strains, conidial tolerances to UV-A and UV-B were reduced by 16.8% and 45.4% for Δ*BbSod2*, 18.7% and 44.7% for Δ*BbSod3*, and on average 33.7% and 63.8% for the RNAi mutants (Fig. 5g), respectively. These data indicated that the effects of BbSod2 and BbSod3 on conidial tolerance to either UV irradiation were additive.

However, conidial tolerance to the high temperature of 45°C did not differ significantly among all the tested strains (*F*_7,16_ = 1.2, *P* = 0.35), as indicated by their median lethal times (LT_50_s) of 51.7–55.9 min under the thermal stress (Fig. 5f).

### Conidial virulence

In the bioassays of *M. persicae* apterae topically inoculated by the standardized spray of 1 ml conidial suspension in the spray tower, the tested strains differed significantly in LT_50_ (*F*_5,17_ = 61.8, *P*<0.0001), which ranged from 5.0 to 7.4 days (Fig. 5h). The mean LT_50_ of 5.2 (5.0–5.4) days from the control strains were 18.8%, 14.5% and 37.1% shorter than the estimates from Δ*BbSod2*, Δ*BbSod3* and three RNAi mutants, respectively. In other words, the virulence of *B. bassiana* against the aphid species was significantly reduced by the disruption of each gene and much more by the silence of both. Also, the effects of the two MnSODs on the virulence were additive.

### Correlation of phenotypic parameters

The total SOD activities from all the tested strains correlated significantly to the parameters of intracellular ROS level, cellular antioxidative capability, UV resistance and virulence (Table 2; 0.788≤r^2^≤0.997, *P*<0.01), respectively. Such linear correlations were also found between conidial antioxidative capability and UV resistance (0.932≤r^2^≤0.975) or virulence (r^2^≈0.82). These data indicated that the SOD activity of *B. bassiana* to large extent determined the conidial anti-oxidative capability, on which the conidial UV resistance and virulence were dependent.

**Table 2:** Linear correlation of phenotypic parameters measured from the control strains and the knockout and RNAi mutants of BbSod2 and BbSod3.

Linear correlation of dependent versus independent variables	r^2^	*P* value
Intracellular ROS level (RFI) vs. SOD activity (U/mg proteins)	0.997	<0.0001
Tolerance of colony growth to menadione (EC_50_) vs. SOD activity	0.867	0.0008
Tolerance of colony growth to H_2_O_2_ (EC_50_) vs. SOD activity	0.794	0.0030
Conidial germination rate at 0.2 mM menadione vs. SOD activity	0.788	0.0032
Conidial germination rate at 2 mM H_2_O_2_ vs. SOD activity	0.793	0.0030
Conidial tolerance to UV-A irradiation (LD_50_) vs. SOD activity	0.890	0.0004
Conidial tolerance to UV-B irradiation (LD_50_) vs. SOD activity	0.896	0.0004
Conidial infectivity (LT_50_ against aphids) vs. SOD activity	0.909	0.0002
Conidial tolerance to UV-A vs. conidial tolerance to 0.2 mM menadione	0.932	0.0001
Conidial tolerance to UV-B vs. conidial tolerance to 0.2 mM menadione	0.975	<0.0001
Conidial infectivity (LT_50_) vs. conidial tolerance to 0.2 mM menadione	0.824	0.0018
Conidial tolerance to UV-A vs. conidial tolerance to 2 mM H_2_O_2_	0.904	0.0003
Conidial tolerance to UV-B vs. conidial tolerance to 2 mM H_2_O_2_	0.974	<0.0001
Conidial infectivity (LT_50_) vs. conidial tolerance to 2 mM H_2_O_2_	0.817	0.0021

## Discussion

As presented above, the cytosolic BbSod2 contributed much more to the total SOD activity of *B. bassiana* than the mitochondrial BbSod3 under normal growth conditions. All the phenotypic changes caused by the knockout of each manganese-cored enzyme and the RNAi suppression of both enzymes confirmed for the first time that the different MnSODs exerted additive influences on the biocontrol potential of *B. bassiana* by mediating the fungal development, antioxidative capability, UV resistance and virulence.

First of all, the disruption of either *BbSod2* or *BbSod3* resulted in delayed sporulation and reduced conidial yield during normal incubation but little change in colony morphology. The sporulation became more defective when both enzymes were suppressed by 91–97% via hairpin RNAi. For the knockout and RNAi mutants, the defected sporulation was apparently attributed to the drastic reductions of their SOD activities, which are known to be essential for regulating cellular redox balance for fungal development [7]. Previously, the expression of a cytosolic MnSOD was strongly induced upon entry into and during the stationary growth phase of *C. albicans* [5]. A positive correlation was also found between SOD activity and sporulation capacity in some fungi [15], [23]. Our results support the previous observations. Apart from the defected sporulation, the double-gene-silenced mutants showed longer delay in conidial germination than each of the single-gene-disrupted mutants. This could result from the increased levels of their intracellular superoxide anions, which may affect polar growth during the emergence of germ tube [24].

Accompanied by the decreased SOD activities and the increased ROS levels, the knockout and RNAi mutants of *BbSod2* and *BbSod3* became remarkably more sensitive to the oxidants menadione and H_2_O_2_ irrespective of mycelia or conidia, well in accordance with the changes of antioxidative capabilities in the previous null mutants of fungal SODs [12], [15], [25]. However, the two MnSODs in this study were not involved in mediating *B. bassiana* response to hyperosmotic and thermal stresses, a phenomenon different from the confirmed effect of MnSOD on yeast osmosensitivity [8] and fungal heat sensitivity [26]. Previously, functional differentiation was found among different SODs of *A. fumigatus*, in which Δ*sod1* and Δ*sod2* were hypersensitive to high temperature and menadione while Δ*sod3* showed a slight sensitivity to high temperature but Δ*sod4* was lethal [12]. Nonetheless, BbSod2 and BbSod3 were proven to respond specifically to oxidative stress at cellular level and their contributions to the fungal antioxidative capability were additive. The fact that the mycelia and conidia of Δ*BbSod2* were more tolerant to the two oxidants than those of Δ*BbSod3* indicates that mitochondrial BbSod3 contributed more to the fungal antioxidative capability under oxidative stress although cytosolic BbSod2 showed much higher SOD activity under normal conditions. This is further evidenced by the higher upregulation of *BbSod3* versus *BbSod2* in the WT cells treated with oxidants and the higher ROS level in Δ*BbSod3* than in Δ*BbSod2* under oxidative stress. Thus, the effects of the two MnSODs are complementary under different conditions. On the other hand, the NaCl-responsive difference between all mutants and WT could be attributed to the *bar* and *sur* markers used in the mutant constructs, suggesting the existence of MnSOD-independent defense system(s) against hyperosmotic and thermal stresses in *B. bassiana*.

Moreover, conidial UV resistance, an important parameter for the fungal biocontrol potential due to its effect on the field persistence of a fungal formulation, was significantly reduced by the disruption of BbSod2 or BbSod3 and much more by the knockdown of both enzymes. This was in agreement with our previous study in which *B. bassiana* tolerance to UV-B irradiation was enhanced by the overexpression of BbSod2 in the enzyme-absent strain [18]. Interestingly, conidial antioxidative capability, as indicated by germination rates under the oxidative stress of menadione or H_2_O_2_, determined the variability of 97% UV-B resistance and of 90–93% UV-A among the MnSOD mutants in the linear correlations of paired variables, respectively.

More importantly, conidial virulence, another crucial parameter for the biocontrol potential of *B. bassiana*, changed in the same pattern as the UV resistance in our study. The suppression of both BbSod2 and BbSod3 reduced the fungal virulence to *M. persicae* more than the deletion of either enzyme. This is likely due to the fact that the double-gene-silenced mutants lost more antioxidative capability (SOD activity) than each null mutant so that they became less capable of detoxifying superoxide anions from infected host cells. Previously, both cytosolic sod1 and cell-wall-anchored sod5 were found necessary for *C. albicans* to achieve full virulence due to their defensive action against oxidative stress produced by macrophages [13], [27]. The null mutant of *C. neoformans* sod1 also became less virulent due to its greater susceptibility to the oxidative stress from host phagocytes [28]. However, a triple *sod1/sod2/sod3* mutant of *A. fumigates* showed little change in virulence despite its high sensitivity to menadione [12]. With these and our results in mind, we consider that the effect of an SOD on fungal virulence may vary with fungal pathogens and the SOD types.

Conclusively, both cytosolic BbSod2 and mitochondrial BbSod3 protected *B. bassiana* from specific damage by superoxide anions. Since their protective effects were additive, both enzymes to a large degree determined the fungal UV resistance and profoundly affected the fungal virulence. Therefore, the two different MnSODs were co-contributors to the biocontrol potential of *B. bassiana*.

## Materials and Methods

### Microbial strains, cultures, reagents and primers

The wild-type strain *B. bassiana* ARSEF2860 (denoted WT in this report) from the RW Holley Center for Agriculture and Health (Ithaca, NY) was grown at 25°C on SDAY plates. *Escherichia coli* DH5α and BL21 (DE3) from Invitrogen (Shanghai, China) were cultured in Luria-Bertani medium for routine vector propagation. *Agrobacterium tumefaciens* AGL-1 was cultured in YEB broth [29] at 28°C for fungal transformation.

The reagents and kits used in the study included restriction endonucleases from New England Biolabs (Beijing, China), La*Taq* DNA polymerase, RNAiso Plus Kit, PrimeScript® RT reagent Kit, and SYBR® Premix Ex Taq™ from TaKaRa (Dalian, China), MitoTraker® Red CMXRos (a mitochondrion-selective probe) from Invitrogen (Shanghai, China) and the fluorescence probe dihydroethidium (DHE) from Beyotime (Jiangsu, China). All chemicals were of analytical purity.

All designed primers are listed in Table 1.

### Cloning of *BbSod3*

The mitochondrial MnSOD gene *BbSod3* was cloned using the previous method for cloning the cytosolic MnSOD gene *BbSod2* from the same WT [18]. Briefly, degenerate primers (Sod3-F/R) were designed in terms of the conserved regions of six mitochondrial MnSOD genes from *A. nidulans*, *Penicillium marneffei*, *Pyrenophora tritici*, *Cordyceps militaris*, *Coccidioides immitis* and *Neurospora crassa* (GenBank IDs: AAF66995, XP_002151294, XP_001931916, AAO47725, XP_001247816 and EAA30249), respectively. Fungal genomic DNA was prepared and total RNA was extracted with the TRIZOL® kit (Invitrogen). A domain fragment of the target gene was amplified via degenerate PCR and the coding region was obtained by two rounds of PCR walking using DNA Walking SpeedUp™ Premix Kit-II (Seegene, Seoul, Korea). Its flanking regions were amplified by PCR walking with nested primers Sod3-upR1/R2 and Sod3-dnF1/F2. All the PCR products were cloned into pGEM-T Easy from Promega (Madison, MI) for sequencing at Invitrogen. The full-length *BbSod3* sequence was finally cloned by PCR with Sod3-F1/R1 primers designed based on ATG- and TGA-neighboring sequences.

### Characterization of BbSod3

The protein sequence deduced from the cloned gene *BbSod3* was compared with those of the referenced fungal MnSODs in the NCBI protein database through online Blast (http://www.ncbi.nlm.nih.gov/Blast.cgi/) and Pfam (http://pfam.sanger.ac.uk/) analyses. Both mitochondria-targeting probability and 5′ transit-leading signal of the deduced BbSod3 were predicted using the software MitoProt II 1.0a4 [30].

The predicted N-terminal mitochondria-targeting signal of 34 amino acids for BbSod3 was amplified from the WT cDNA with SigGFP-F1/R1 primers and fused to the open reading frame (ORF) of the enhanced green fluorescence protein gene *eGFP* (GenBank ID: U55763) amplified from pEGFP-C1 (BD Biosciences Clontech, CA) with SigGFP-F2/R2. The fusion was achieved by means of splicing-by-overlap extension (SOE) [31] and the product *BbSod3signal::eGFP* was amplified with SigGFP-F1/R2. The fused gene was digested with *Bgl*II and cloned into *Bam*HI-linearized and dephosphorylated pAN52-1N bearing *A. nidulans* P*gpdA* and T*trpC*, forming pAN52-sigGFP. Subsequently, the phosphinothricin (PPT) resistance gene *bar* (as selective marker) from pAN52-bar [32] was inserted into pAN52-sigGFP linearized with *Xba*I and dephosphorylated, yielding pAN52-sigGFP-bar for fungal transformation. This new plasmid was linearized with *Bgl*II and finally integrated into the WT strain using the method of blastospore transformation [32].

The expression of the fusion gene in a positive transformant (SigG6) was verified for the intracellular localization of the targeted protein by the fused BbSod3 signal peptide. Aliquots of 100 µl of 10^7^ conidia/ml suspension were mixed with 20 ml of Sabouraud dextrose broth (SDB) and incubated by shaking (150 rpm) at 25°C for 2 days. The resultant mycelia were stained with 500 nM MitoTracker Red CMXRos and examined for the presence of red and green fluorescence under a laser-scanning confocal microscope (Carl Zeiss, Germany). The same images, labeled with the stain and the expressed eGFP, in two channels were collected and then overlapped for comparison using Zeiss LSM5 image browser. A transgenic strain expressing the signal-free eGFP was used as a control.

Native polyacrylamide gel electrophoresis (Native PAGE) was performed to detect isoenzymatic SOD activities in the total protein extract from the WT culture as described previously [18]. The SOD profiles were visualized on the gels stained with nitroblue tetrazolium (NBT). Different SODs were differentiated by staining the gels after 20 min incubation at 25°C in 50 mM potassium phosphate buffer (pH 7.8) or in the buffer containing 2 mM KCN or 5 mM H_2_O_2_. The differentiation was based on the inhibitory effects of KCN and H_2_O_2_ on the activities of Cu/ZnSOD and FeSOD, respectively, and no inhibitory effect of both chemicals on MnSOD activity [33].

For Western blotting analysis, BbSod3 was expressed in transgenic *E. coli* BL21 (DE3) and purified by one-step nickel affinity chromatography as described previously for purified BbSod2 [18]. The purified enzyme fractions of BbSod2 and BbSod3 were separately dialyzed with dd-H_2_O, vacuum-dried overnight at room temperature and standardized to 1 mg/ml saline. The polyclonal antibodies anti-BbSod2 and anti-BbSod3 were prepared with the saline samples through professional service at the authorized Experimental Animal Center of Zhejiang Chinese Medical University (Hangzhou, China). The crude protein extract from the WT culture was electrophoretically transferred from non-denaturing gel onto PVDF membrane for Western blotting using the kit of ProtoBlot alkaline phosphatase system (Novagen, Madison, WI). Two blots were probed with 1000× dilution of anti-BbSod2 and anti-BbSod3 and visualized with goat anti-rabbit IgG-alkaline phosphatase conjugate (Novagen) to distinguish the isoenzymatic SOD bands on the NBT-stained gels.

### Disruption and complement of *BbSod2* and *BbSod3*

*BbSod2* (GenBank ID: GU122855) and *BbSod3* were disrupted and complemented through homologous replacement and random integration. The P*trpC* and *bar* elements were amplified from pCB1003 and pAN52-bar [32] using paired primers PtrpC-F/R and Bar-F/R, respectively, and fused using the primers PtrpC-F/Bar-R with several restriction sites introduced to both ends. The P*trpC*-*bar* cassette was cloned into the *Hin*dIII-*Bgl*II sites of pCAMBIA-0380 (CAMBIA, Canberra, Australia; p0380 herein) to generate p0380-bar. The 3′ and 5′ ends of *BbSod2* were cloned by PCR with paired primers R1/R2 and L1/L2 using La*Taq* DNA polymerase and inserted respectively into the *Xho*I/*Spe*I and *EcoR*I/*Bam*HI sites of p0380-bar, forming the plasmid p0380-bar-Sod2d for *BbSod2* disruption. Similarly, p0380-bar-Sod3d was constructed for *BbSod3* disruption by cloning the 3′ and 5′ ends of *BbSod3* with paired primers R3/R4 and L3/L4.

To complement each gene, *Magnaporthe grisea* acetolactate synthase gene (*sur*) cassette was amplified from pCB1536 [34] with primers Sur-F/R and inserted into *EcoR*I-*Bam*HI sites of p0380-bar as the second marker, forming p0380-sur-bar. A gateway fragment was cut from pGK02 [35] with *Xba*I/*Hin*dIII and inserted into the enzyme sites of p0380-sur-bar to replace *bar*, yielding p0380-sur-gateway. The full-length fragments of *BbSod2* and *BbSod3* with the untranslated regions (UTR) of 2551 and 1530 bp at the 5′ ends and 989 and 1482 bp at the 3′ ends were amplified from the WT genomic DNA using the La*Taq* polymerase and paired primers Sod2C-F/R and Sod3C-F/R, respectively. Each amplified fragment was used to exchange for the gateway fragment in the plasmid under the action of Gateway® BP ClonaseTM II Enzyme Mix (Invitrogen), yielding p0380-sur-Sod2c or p0380-sur-Sod3c, which was transformed into *E. coli* DH5α for propagation and used for complementing each target gene in the knockout construct.

All disruption and complement plasmids were separately mobilized into *A. tumefaciens* AGL-1 and subsequently transformed into WT or the knockout mutant of each gene using the previous protocol [28], [36]. The disruption and complement mutants of *BbSod2* and *BbSod3* were screened based on the *bar* and *sur* resistance to PPT (200 µg/ml) and chorimuron ethyl (10 µg/ml) included in M-100 plates [29], respectively, and detected for the presence or absence of each target gene by PCR with the primers I1/I2 and I3/I4. The positive mutants, i.e., Δ*BbSod2*, Δ*BbSod2*/*BbSod2*, Δ*BbSod3* and Δ*BbSod3*/*BbSod3*, were verified by Native PAGE for the SOD profiling changes as above.

### Constructing the RNAi mutants of *BbSod2::BbSod3*

To construct the RNAi plasmids for silencing simultaneously *BbSod2* and *BbSod3*, the ORF (630 bp) of *BbSod2* was fused to the partial *BbSod3* ORF fragments of 304 and 298 bp (free of the signal peptide-coding sequence) using the SOE method with six pairs of sense and antisense primers, yielding the *BbSod2::BbSod3* genes *F1* and *F2*, respectively. The RNAi cassettes were designed as inverted *F1* or *F2* repeats of ∼900 bp separated by a 320-bp loop, which was the first intron of the Cu/ZnSOD gene *BbSod1* (GenBank ID: GQ304735) reported previously [17] and was amplified from the genomic DNA with I-F/I-R primers. The loop and *F1* or *F2* were sequentially inserted into the corresponding enzyme sites of pAN52-1N, forming the hairpin RNAi plasmid pAN52-F1silence or pAN52-F2silence. The *bar* marker was then inserted into each plasmid for final integration into the WT genome using the method of blastospore transformation [32].

### Assay for transcript levels of two target genes in RNAi mutants and WT

Ten RNAi mutants taken from PPT-inclusive Czapek's plates were assayed for the transcript levels of their *BbSod2* and *BbSod3* by qRT-PCR Total RNA was extracted from the cultures of each mutant grown for 3 days on the plates of Sabouraud dextrose agar plus 1% yeast extract (SDAY) at 25°C using the RNAiso Plus Kit. All the cultures were initiated by spreading 100 µl of 10^7^ conidia/ml suspension onto cellophane attached to the plates. The quantity and purity of RNA samples were determined through spectrophotometry. The cDNA was reversely transcribed from the total RNA with the PrimeScript® RT reagent Kit at 37°C for 15 min and terminated at 85°C for 5 s. The cDNA product was diluted in DEPC water (1∶20). The transcript levels of *BbSod2* and *BbSod3* relative to that of 18S rRNA in each cDNA sample were assessed via qRT-PCR with SYBR® Premix Ex Taq™ and paired primers in Table 1. The qRT-PCR started from polymerase activation of 30 s at 95°C, followed by 40 cycles of 30 s at 95°C and 34 s at 60°C. The transcript level of each gene in the cDNA was estimated using the 2^−ΔΔCt^ method [37]. The ratio of each gene transcript in an RNAi mutant over that in WT was defined as the relative transcript level. Three mutants (F1-4, F2-2 and F2-4) with maximal silence of both target genes were selected for the following experiments.

Additionally, the same protocol was used to monitor the temporal transcript patterns of *BbSod2* and *BbSod3* during the 7-day growth of the WT strain on SDAY at 25°C or examine their transcript changes in the WT cultures grown for 3 days on SDAY under the oxidative stress of 0.2 mM menadione or 4 mM H_2_O_2_.

### Assay for overall SOD activity

Total protein extracts from 0.5 g aliquots of fresh mycelia from Δ*BbSod2*, Δ*BbSod3*, three RNAi mutants and three control strains (WT, Δ*BbSod2*/*BbSod2* and Δ*BbSod3*/*BbSod3*) were prepared by suspending ground samples in 50 mM phosphate buffer (pH 7.4). After centrifugation at 16,000×g (20 min at 4°C), the supernatant of each sample was assayed for overall SOD activity based on the inhibition of spontaneous autooxidation of pyrogallol [38]. The protein concentration (mg/ml) was assessed using bovine serum albumin as a standard. One unit of SOD activity was defined as the SOD amount required to inhibit 50% pyrogallol autoxidation rate and expressed as U/mg proteins.

### Assay for intracellular ROS levels

The intracellular ROS levels of all the mutants versus WT were assayed using the fluorescence probe DHE [39]. Briefly, mycelia and blastospores harvested from SDB cultures shaken for 2 days at 25°C were transferred to NLM (4% glucose, 0.4% NH_4_NO_3_, 0.3% KH_2_PO_4_ and 0.3% MgSO_4_) for 24 h incubation, yielding thin-walled, unicellular blastospores [32]. Harvested by filtration, the blastospores were resuspended in 1 ml NLM (standardized to 10^7^ cells/ml) containing 0.2 mM menadione, 2 mM H_2_O_2_ or no oxidant for 1 h incubation at 25°C. Washed twice with with 0.1 M Tris-HCl buffer (pH 8.0), the stressed and unstressed cells were separately stained with 5 µM DHE at 37°C for 30 min. The stained cells were washed twice with the buffer and resuspended in 1 ml of 0.1 M Tris-HCl (pH 8.0). The fluorescence intensity of the stained cells in the suspension was immediately assessed on FC500 Flow Cytometer (Beckman Coulter, CA) at the excitation/emission wavelengths of 510/615 nm. Relative fluorescent intensity (RFI) indicating the ROS level in the cells was estimated as (F-F_t_)/(F_w_-F_0_), where F and F_t_ were the readings from the stained and unstained cells of a given mutant, and F_w_ and F_0_ were the readings from the WT counterparts.

### Assay for sporulation capacity and conidial quality

Two knockout mutants (Δ*BbSod2* and Δ*BbSod3*) and three RNAi mutants (F1-4, F2-2 and F2-4) were assayed together with the control strains in triplicate experiments to measure phenotypic parameters below.

To assay sporulation capacity, aliquots of 100 µl suspension (10^7^ conidia/ml; the same below unless mentioned otherwise) were evenly smeared on SDAY plates and incubated for 7 days at 25°C and 12:12 h (light : dark cycle). From the third day onwards, 5-mm-diameter disks were cut daily from the plates. Conidia on the colony discs were washed off into 1 ml of 0.02% Tween 80 by ∼10 min supersonic vibration, followed by filtration through four layers of lens tissues to remove mycelial debris. The concentration of the conidial suspension was assessed using microscopic counts with a hemocytometer and then converted to the number of conidia/cm^2^ colony.

To assay conidial germination rate, aliquots of 50 µl conidial suspension were spread onto the plates of germination medium (GM: 2% sucrose and 0.5% peptone and 1.5% agar), followed by 24 h incubation at 25°C and 12:12 h. From 8 h onwards, percent germination on each plate was assessed every 4 h using three microscopic counts (≥100 conidia per count).

### Assay for colony tolerances to oxidative and hyperosmotic stresses

Mycelial mass discs (5 mm diameter) were cut from the cultures grown for 3 days at 25°C on cellophane attached SDAY plates, on which the aliquots of 100 µl conidial suspension were evenly spread to initiate the cultures. The discs were transferred onto the plates (90 mm diameter) of 1/4 SDAY (SDAY amended by reducing all nutrients to 1/4 of normal proportion) supplemented with 0–8 mM menadione, 0–60 mM H_2_O_2_ or 0–1.5 M NaCl, followed by 5-day incubation at 25°C. The sizes of all colonies under the stresses of oxidation and hyperosmolarity were daily assessed by cross-measuring their diameters during growth.

### Assay for conidial tolerances to oxidation, UV irradiation and heat

The conidial tolerance of each strain to oxidative stress was assayed by spreading the aliquots of 100 µl conidial suspension onto the GM plates containing 0.2 mM menadione or 2 mM H_2_O_2_. After 24 h incubation at 25°C, percent germinations were assessed as mentioned previously.

The UV-B and UV-A stress assays of each strain were performed using a previous method [40]. Briefly, 10 µl aliquots of the suspension of 10^6^ conidia/ml germination broth (GB: agar-free GM) were centrally spotted onto marked area (∼10 mm diameter) of glass slides and then exposed to the irradiation of the weighted UV-B wavelength of 312 nm (280–320 nm) at the gradient doses of 0.1–0.8 J/cm^2^ or the weighted UV-A wavelength of 365 nm (320–400 nm) at 6–30 J/cm^2^ in Bio-Sun^++^ chamber (Vilber Lourmat, Marne-la-Vallée, France). The wavelength specific irradiations were emitted from two 30W fluorescent tubes above a sample tray and adjusted automatically for error control of ≤1 µJ/cm^2^ at each dose (the user's guide). After exposure, the slides were removed from the tray and incubated at 25°C and 12:12 h under saturated humidity. After 24-h incubation, the marked areas were stained with cotton blue and examined for the counts of germinated and ungerminated conidia under a microscope.

To assay conidial thermotolerance, aAliquots of 1 ml conidial suspension in glass tubes were exposed to hot water bath at 45°C for up to 90 min as described previously [18]. During the exposure, 100 µl aliquot was pipetted from each stressed tube every 15 min and released into 1 ml GB, followed by 24-h shaking for germination. Germinated and ungerminated conidia were counted with a hemocytometer to determine the percent germination of each strain after a given time of thermal stress.

### Assay for conidial virulence

Batches of 30–40 apterous adults (≤24 h after last ecdysis) of green peach aphid *Myzus persicae* on cabbage leaf discs, which were prepared with hairy roots growing from edges into agar plates in Petri dishes (9 cm diameter) as described previously [41], were separately sprayed with 1 ml of conidial suspension of each strain using an automatic Potter Spray Tower (Burkard Scientific Ltd, Uxbridge, UK). The same-volume spray of 0.02% Tween 80 (used for suspending conidia) was included as a control of each assay. All sprayed aphids were reared *in situ* for 8 days at 25°C and 12:12 h and examined daily for mortality records.

### Data analysis

The ratio of fungal viability (conidial germination or colony growth) under a given stress over that in the unstressed control was defined as survival index (*I*_s_). The *I*_s_ trends over the concentrations (*C*) of menadione, H_2_O_2_ or NaCl, the doses (*D*) of the UV-B or UV-A irradiation, and the time lengths (*T*) of the heat stress at 45°C or of incubation for germination were separately fitted to the equation *I*_s_ = 1/[1+exp(*a*+*bx*)], where *x* represents *C*, *D* or *T*, and *a* and *b* are the parameters to be estimated for the *I*_s_-*x* relationships. When *I*_s_ = 0.5, the fitted equations provided solutions (−*a*/*b*) for the time length required for 50% conidial germination (GT_50_), the effective concentration of a stress-specific chemical agent to suppress 50% colony growth (EC_50_), and the median lethal dose of UV-A or UV-B irradiation (LD_50_) and the median lethal time of the heat stress (LT_50_) against conidia. Each solution was a quantitative index for the observed phenotype or the tolerance of each fungal strain to the specific stress. Aphid mortality data over post-spray days were subjected to probit analysis, generating the LT_50_ estimates for the tested fungal strains to kill 50% aphids under the standardized spray. Observations or phenotypic parameters were subjected to one-factor (strain) analysis of variance. Linear correlations were performed to reveal possible relationships between paired phenotypic variables showing significant changes among the tested fungal strains.
